# Left minimally invasive esophagectomy in a patient with synchronous esophageal and lung cancers: Case report

**DOI:** 10.1097/MD.0000000000009173

**Published:** 2018-01-12

**Authors:** Baihua Zhang, Junliang Ma, Xinjian Yan, Xu Li, Qin Xiao, Wenxiang Wang, Yong Zhou

**Affiliations:** a The 2nd Department of Thoracic Surgery; b Key Laboratory of Translational Radiation Oncology, Department of Radiation Oncology, Hunan Cancer Hospital, The Affiliated Cancer Hospital of Xiangya School of Medicine, Central South University, Changsha, China.

**Keywords:** esophageal cancer, left-sided approach, lobectomy, lung cancer, minimally invasive esophagectomy

## Abstract

**Rationale:**

Minimally invasive esophagectomy (MIE) have been increasingly used and are regarded as suitable alternatives to open esophagectomy. However, few previous reports described minimally invasive esophagectomy using a left-sided approach.

**Patient concerns and diagnoses:**

A 71-year-old man was admitted to our hospital because of progressive dysphagia. Synchronous double primary thoracic esophageal and left lung cancers were considered before the operation.

**Interventions and outcomes:**

A lobectomy and MIE, via a left video-assisted thoracoscopic approach, was performed. Preparation of a gastric conduit and an intra-abdominal lymphadenectomy were completed by laparoscopy and a cervical anastomosis was made. In addition, a cervical mediastinoscopy was performed to dissect the lymph nodes along the bilateral recurrent laryngeal nerves. No postoperative complications were observed. The patient achieved a favorable short-term outcome.

**Lessons:**

This is the first report of a patient with synchronous esophageal and left lung cancers treated with minimally invasive resection via left thoracoscopy, laparoscopy, and cervical mediastinoscopy. Our results showed that the left MIE approach in combination with cervical mediastinoscopy is potentially most appropriate for some esophageal cancer patients, when the right MIE approach is not applicable in certain conditions.

## Abstract 2

Supplemental Digital Content is available in the text

## 1 Introduction

Surgical treatment remains the primary curative option for resectable esophageal cancer. However, several recent studies demonstrated that minimally invasive Ivor-lewis and Mckeown approaches to esophagectomy might achieve lower morbidity, greater short-term benefits, and similar long-term survival rates, compared with open surgery.^[1,2]^ As a result, such minimally invasive approaches to esophagectomy have been increasingly used and are regarded as suitable alternatives to open esophagectomy.

To the best of our knowledge, current minimally invasive esophagectomy (MIE) is performed most often via right-sided thoracoscopy, to afford better exposure of the thoracic esophagus. Few reports, therefore, described MIE via the left-sided approach. In this report, we treated a patient with thoracic esophageal cancer and a synchronous pulmonary nodule, located in the left superior lobe, entirely with MIE and lobectomy, using a left thoracoscopic and laparoscopic surgical approach. Additionally, video-assisted mediastinoscopy, via the neck, was performed to dissect the lymph nodes along the bilateral recurrent laryngeal nerves.

## 2 Case presentation

**Figure 1:**
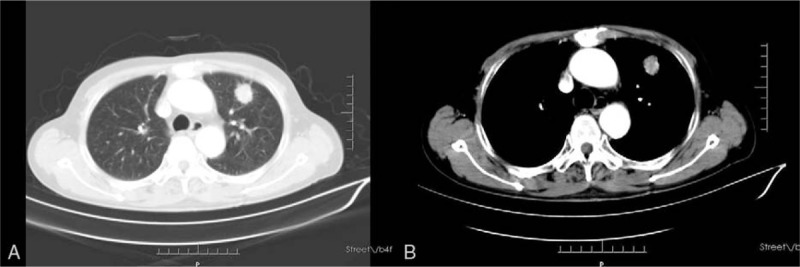
A, B. Chest and upper abdominal computed tomography (CT) showed a solitary nodule, measuring 2.3 cm in diameter, in the left superior lung lobe.

**Figure 2:**
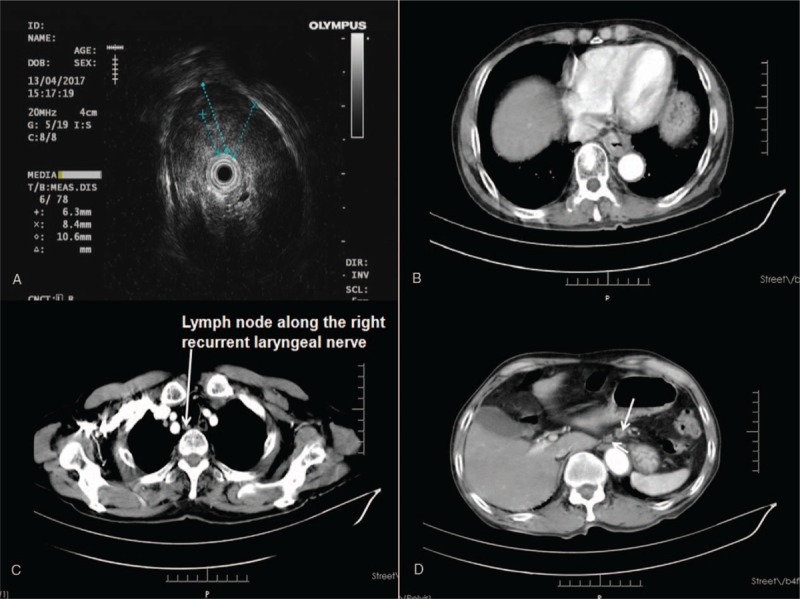
A. By endoscopic ultrasound, the esophageal tumor was staged as T3N1. B. CT scan showed wall thickening in the lower esophagus. C. A 5 mm lymph node was detected with the suspicion of metastasis along the right recurrent laryngeal nerve. D. Lymph nodes, >10 mm in diameter, were detected around the left gastric artery and gastric cardia. CT = computed tomography.

A 71-year-old man was admitted to our hospital because of progressive dysphagia, for >5 months, and a slowly enlarging nodule in the left lung, detected for 2 months. The patient was diagnosed with a squamous cell carcinoma in the lower third thoracic esophagus and a nodule in the left superior lung lobe. These were considered to be synchronous double primary lesions, based on the imaging characteristics. Chest and upper abdominal computed tomography (CT) showed wall thickening in the lower esophagus, accompanied by a solitary nodule, 2.3 cm in diameter, in the superior lobe of the left lung (Figs. 1 and 2). Lymph nodes >10 mm in diameter were detected around the left gastric artery and gastric cardia. Additionally, a 5 mm lymph node was found with the suspicion of metastasis along the right recurrent laryngeal nerve. By esophagogastroscopy, an ulcerative mass, 35 to 40 cm from the upper incisors was identified. Using endoscopic ultrasound, the esophageal tumor was staged as T3N1. No distant metastases were detected in this patient prior to treatment.

**Figure 3:**
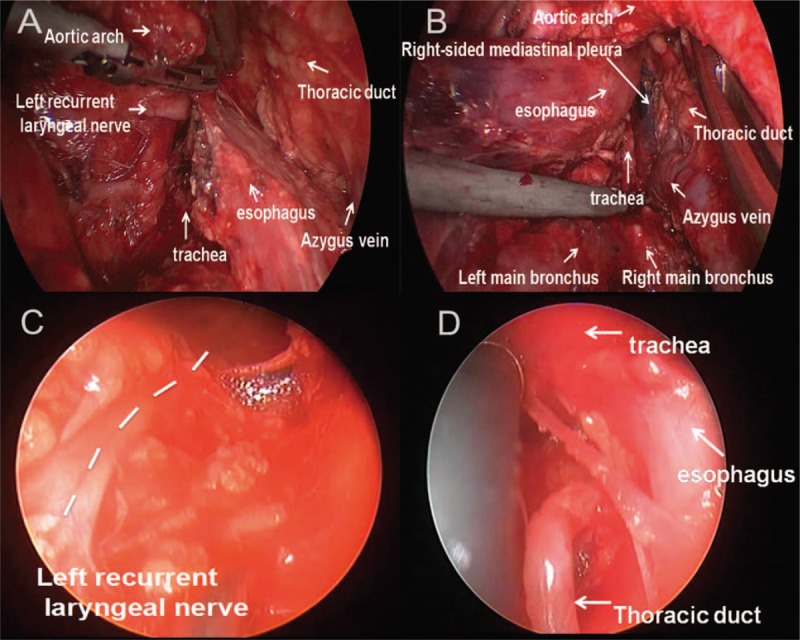
A, B. Anatomy behind the aortic arch via left thoracoscopy. C, D. The left recurrent laryngeal nerve and thoracic duct were exposed via cervical mediastinoscopy.

The patient refused any treatment, except surgical resection, because of his advanced age. Therefore, an MIE and lobectomy was performed, via a left video-assisted thoracoscopic surgical approach. First, we performed thoracoscopic lobectomy and thoracic esophageal mobilization in the right lateral position (Supplemental video 1). The operator was positioned in front of the patient. Based on our prior experience, a 2 cm incision at the 8th intercostal space, along the middle axillary line, was selected as the observation port. The principal operating port, with a 3 cm access incision, was made at the 5th intercostal space, before the anterior axillary line. Another 2 cm assisting operating port was located at the 8th intercostal space, along the posterior axillary line. Dissection of the left superior lung lobe was completed at the beginning of surgery. The patient was then turned to a right lateral-prone position and the posterior mediastinum was exposed. The thoracic esophagus behind the aortic arch and the lower part were readily mobilized. Lymph nodes along the left recurrent laryngeal nerve and thoracic esophagus were also dissected (Fig. 3A and B).

After that, laparoscopy was completed, using a standard procedure with the patient in the supine position, as previously reported.^[3]^ Stomach mobilization and upper abdominal lymphadenectomy were completed. The gastric lesser curvature was then trimmed with a linear endostapler and the gastric tube conduit was made extracorporeally. A cervical collar incision was made in the suprasternal fossa and elongated along the inner edge of the left sternocleidomastoid muscle (supplemental video 2). The bilateral recurrent laryngeal nerves were exposed under direct vision, as for thyroid surgery. The mobilization of the upper third of the esophagus and the lymphadenectomy along the bilateral recurrent laryngeal nerves were completed (Fig. 3C), via video-assisted mediastinoscopy, as previously described.^[4,5]^ The thoracic duct was exposed and ligated with a Hem-o-lock clip (Fig. 3D). The gastric conduit was pulled up, using a posterior mediastinum approach, and a triangular mechanical esophagogastric anastomosis was made in the neck to avoid lethal anastomotic leakage.

The length of the postoperative hospital stay was 11 days, with no postoperative complications observed. The final pathological examination revealed a moderately differentiated squamous cell carcinoma invading the esophageal adventitia (pT3). A total of 40 nodes were dissected, with 4 positive nodes, along the left gastric artery and gastric cardia, were detected (N2). The pulmonary nodule in the left superior lobe was identified as a moderately differentiated adenocarcinoma with no lymphatic metastasis (pT2N0M0).

## 3 Discussion

Few cases described application of MIE via left-sided thoracoscopy, except in a few patients with situs inversus totalis.^[6–8]^ However, because the right-sided arotic arch obstructed dissection of the proximal intrathoracic esophagus in those cases, as a mirror image approach compared with standard MIE techniques, a left thoracoscopy was favored for resection. As a result, application of MIE using the real left-sided approach has been seldomly reported before.

In our patient, the existence of synchronous double primary esophageal and left lung cancers was considered before the operation. In previous studies,^[9,10]^ traditional open resection was the principal method of curative treatment for synchronous multiple primary cancers involving esophageal cancer. However, considering this patient's age and emaciation, a surgical approach, such as minimally invasive resection, was a favorable choice to decrease the risk of postoperative morbidity. Therefore, a left MIE and lobectomy were preferable for achieving an intention-to-cure resection.

In our procedure, we first resected the superior lobe of the left lung and mobilized the middle and lower thoracic esophagus via left-sided thoracoscopy, as performed in open surgery. Furthermore, a lymphadenectomy in the middle and lower mediastinum could also have been readily performed. However, exposing the upper thoracic esophagus and performing lymphadenectomy in the upper mediastinum were hindered by presence of the aortic arch. Therefore, video-assisted cervical mediastinoscopy, rarely used in esophagectomy, was introduced to treat this case.

Some authors proposed that video-assisted mediastinoscopic lymphadenectomy could be a clinically feasible supplementary procedure in minimally invasive radical resection of lung cancer,^[11]^ because mediastinoscopy can improve lymphadenectomy in the upper mediastinum.^[5,11]^ Witte et al^[5]^ and Wang et al^[4]^ reported that lymph nodes along the recurrent laryngeal nerves could be dissected and sampled using mediastinoscopy. Wang et al^[4]^ demonstrated that, in patients with T1 esophageal cancer, mediastinoscopy-assisted esophagectomy achieved short-term and long-term outcomes similar to those of thoracoscopic esophagectomy. Consistent with this, our case identified transcervical extended mediastinoscopic lymphadenectomy as a new surgical procedure allowing for dissection of the upper third thoracic esophagus and complete lymphadenectomy in the upper mediastinum from the neck.

To the best of our knowledge, this is the first report of a patient with synchronous esophageal and left lung cancers treated with minimally invasive resection via left thoracoscopy, laparoscopy, and cervical mediastinoscopy. This patient represented a particularly unique case because he presented with synchronous esophageal and left lung carcinomas and, also, refused any treatment except surgical resection. Our results showed that the left MIE approach in combination with cervical mediastinoscopy was surgically feasible. It is potentially most appropriate for some patients, when the right MIE approach is not applicable in certain conditions, such as atresia of the right thoracic cavity.

## Supplementary Material

Supplemental Digital Content

## Supplementary Material

Supplemental Digital Content
